# Hysteroscopy for training residents using uterine post-hysterectomy specimens with a mobile hysteroscope

**Published:** 2020-05-07

**Authors:** I Chatzipapas, N Kathopoulis, A Protopapas, D Loutradis

**Affiliations:** 1^st^ Department of Obstetrics and Gynecology, University of Athens, Alexandra Hospital, Athens, Greece.

**Keywords:** Mobile hysteroscopy, hysteroscopy, hysterectomy, training, simulation

## Abstract

**Background:**

Training in hysteroscopy can be challenging, especially in conscious women as an office procedure.

**Objective:**

To develop a realistic hysteroscopy training model for residents using human uteri.

**Methods:**

Human uterine specimens were acquired immediately after hysterectomy, before they were sent for histological analysis and were used as a training model for hysteroscopy.

**Results:**

We describe this new technique, which we have used for one year in our resident training programme. Each resident performs at least 20 simulated diagnostic hysteroscopies in extirpated uteri, before performing procedures on women in the operating room.

**Conclusions:**

Simulating hysteroscopy on human uterine models offers a novel and realistic way of training novices prior to conducting procedures under supervision on live patients.

**What is new:**

This is a novel model for training and offers a much more realistic training opportunity.

## Introduction

Hysteroscopy is a valuable tool in gynaecological practice that provides direct visualisation of the uterine cavity. Hysteroscopy can be used for diagnostic and therapeutic purposes. Uterine bleeding, infertility, endometrial ablation and removal of intrauterine pathology (polyps, myomas) are some of the common indications for this procedure. Hysteroscopic procedures, although relatively safe, may result in serious complications related to either suboptimal surgical skills or prolonged operative times (Munro et el., 2015). The gradual exposure of a surgeon to laparoscopic surgery where junior and senior surgeons can collaborate also partly exists in hysteroscopy training. Since hysteroscopy is an easier and more limited skill to learn than laparoscopy, progress is faster.

Training in hysteroscopy may be challenging. Both gynaecologists and residents acknowledge the importance of aquiring basic endoscopic ability before entering the operating room to perform procedures on live patients. Specifically, to successfully conduct hysteroscopic surgery, trainees must aquire proficiency in operating in two dimensions, using a fixed access point and with a limited range of movements. Thus, the surgeon has to master specific psychomotor skills alongside excellent hand-eye coordination in order to be safe and effective (Campo et al., 2016). On the other hand, ethical concerns challenge the traditional model of learning by experience with real patients and training has been transferred outside of the operating theatre (Papanikolaou et al., 2019).

For these reasons, several types of hysteroscopic simulation models have been reported. Animal organs, fruits and vegetables, synthetic uteri and finally virtual reality simulators are some of the models currently reported as hysteroscopic knowledge and skill enrichment methods. Cost, training capacity, realism, model preparation and storage are some of the issues that a training centre must deal with when choosing the proper model to train the residents (Gambadauro et al., 2018).

Mobile technology, on the other hand, has already been used in healthcare settings to facilitate fiberoptic intubation, endoscopic urological evaluation and ventricular catheter placement. Our department has developed a portable hysteroscopy setup with the utilisation of an iPhone 6s smartphone, a specially designed adaptor, and a portable light source. The mobile phone is transformed into a completely mobile hysteroscopic viewing system with complete portability (Chatzipapas et al., 2018).

In order to overcome these problems and provide the residents with more realistic training before entering the operating room, our department has developed a portable model for the training of diagnostic hysteroscopy using extirpated human uterine specimens immediately following hysterectomy, before they are sent to the pathology department for analysis.

## Description of the hysteroscopic simulation

**Figure 1:**
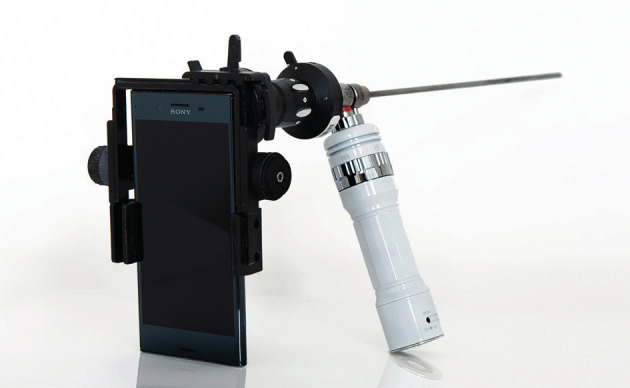
— Mobile hysteroscope

Hysteroscopies were performed with a rigid 300, 2,9mm HOPKINS hysteroscope fitted with a 4 mm outer sheath (Karl Storz, Tuttingen, Germany). The portable setup was created by coupling the hysteroscope via a commercially available adaptor with an iPhone 6s (Apple Inc., Cupertino, CA) or Sony Xperia XZ Premium (Sony Co., Tokyo, Japan). The adaptor used was a ClearSCOPE (Clearwater Clinical Inc., Ottawa, Canada). The light source used was a portable and rechargeable light-emitting diode cold light source (Tonglu Kanger Medical Instrument Co., Hongzou, Zhejiang, China) (Figure 1). We used normal saline with standard intravenous tubing as the distension medium. The saline bag was placed into a pressure cuff under 120-150mmHg.

The uterine model used in our study is a human uterus extracted after hysterectomy. The uteri are transferred after the operation for gross examination to the pathology department, in special containers labelled with patient information, without formalin or any solution . At Around 12:00 noon on average three or four specimens are available and we transport them to a special lab where we perform a hysteroscopy as described.

The hysteroscope is guided to penetrate the external os under direct vision, avoiding touching the walls of the cervix. The distension medium permits distension of the cervical canal, and the internal os must always be kept at six o’clock on the screen for correct orientation of the telescope. Once the isthmus is passed the systematic evaluation of the uterine cavity follows in order to complete the exercise. Uterine fundus, right ostium, left ostium, anterior and posterior walls are observed in that order. Every single resident practices for ten minutes on each uterine model.

**Figure 2:**
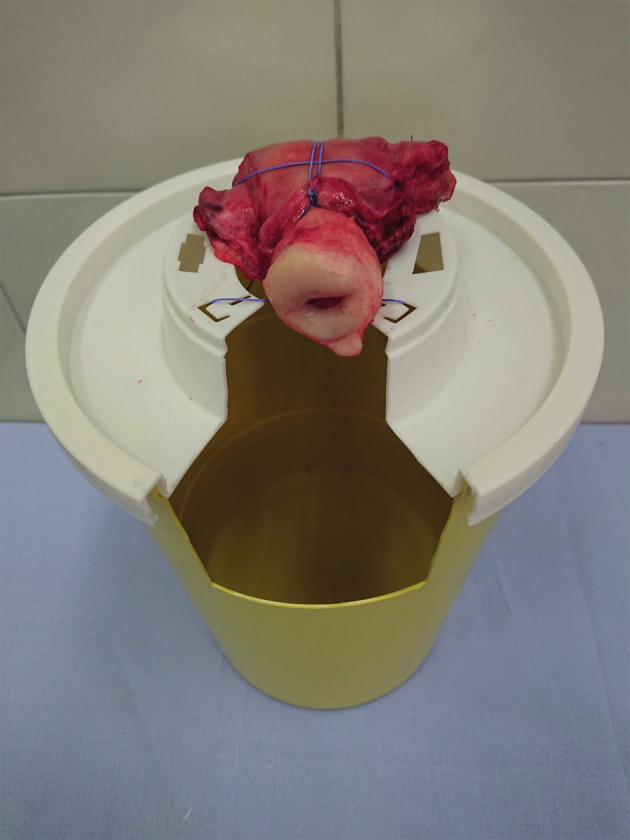
— Uterus box base

The simulation model utilises a uterus box base comprising of a hospital waste safe box, made from strong plastic complete with a plastic lid. We created a window by cutting the lid and part of the box using power rotary tools or a knife. To be stable the box base is one-third filled with water, and the lid is fixed in place. The uterus is cleaned with absorbent paper and stabilised and secured with a suture which passes across the holes of the lid (Figure 2). The lid works as a drip tray, allowing irrigation fluid to be captured throughout diagnostic hysteroscopy. (Video S1, S2 - https://qrco.de/Chatzipapas)

A faculty member supervises a group of three residents in this short course. Three different hysteroscopes (one for each uterine specimen) are used so we can ensure the absence of contamination of the uterine material. At the end of the practice, the specimens are put in formalin solution and returned to the pathology department for further evaluation. If strict timekeeping is observed, there are no concerns from the pathologists regarding degradation risk. Each specimen (one by one) is taken out from the container, used for the hysteroscopy, and is replaced in its box in order to avoid any misidentification risk.

Every specimen is used after the woman has been informed of the process, and all the details have been explained. The specimens come from women operated on for benign pathologies such as myomas, adenomyosis, ovarian cysts and uterine bleeding; malignant conditions are excluded for ethical reasons. This process allows us to obtain the most realistic model for our students’ education without the possibility of damage to the pathology specimen.

We have been using this new teaching technique for a year now in our residency programme. Every day about three or four uterine specimens, obtained from hysterectomies for benign pathology, are available in our department for a short hysteroscopic course. Each resident must perform 20 diagnostic hysteroscopies in this way before entering the operating room to perfrom procedures on real patients. We chose this number based on reports that novices may progress towards competency when exposed to between five and nine repeat camera navigation exercises. Moreover, retention of navigation skills two weeks after baseline training have been reported (Janse et al., 2014; Bajka et al., 2010).

## Discussion

The benefits of using human models to perform hysteroscopy include its realism, widespread availability of specimens and low cost. Most of the hysteroscopy simulation models aim to resemble the circumstances found in real-time hysteroscopy as fully as possible. Reproducible hysteroscopy models may be obtained from fruits and vegetables such as potatoes and butternut pumpkins, which resemble human uteri. Commercial uterine models constructed from latex have also been proposed for this purpose, but all these have the disadvantage of suboptimal realism and lack of anatomic details. On the other hand, virtual reality simulators have positive feedback on realism, training capacity and usefulness, but they have lower ratings for haptic feedback. High fidelity simulation is however, more expensive and not widely available. Using hysterectomy specimens, we managed to obtain the most realistic conditions for residents’ training in a widely accessible way. Haptic feedback is excellent and trainees are challenged to find the path of the uterine cavity and to recognise all anatomic landmarks as in a real procedure. They may repeat the exercise several times with the same specimen. Moreover, the uteri provided from hysterectomies may have pathologic entities such as polyps, fibromas and septum, and residents may have the opportunity to diagnose the pathology.

Another important characteristic of our method is the extremely low cost resulting from the use of the specific model and the new hysteroscopic setup. The hysterectomy specimens have no cost and are advantageous compared to other models on an economic basis. Aside from fruit models that are inexpensive, latex uterine models have significant costs, whereas animal models have additional maintenance preparation and collection expenses, on top of the cost of their acquisition. Finally, virtual reality simulators, with a median cost of $100,000, may be prohibitively expensive for widespread adoption (Jansen et al., 2000). Moreover, our portable hysteroscopic setup has an average cost of $1,699 (telephone, adapter, light source), 25 times lower than the average cost of the standard hysteroscopic tower ($40,000-$50,000). This way hospitals with low financial resources are able to afford a valuable tool for hysteroscopic training and avoid using the standard tower for this purpose, in order to minimize possible damage to their basic equipment. The development of hysteroscopic skills outside the operating room could be beneficial for the healthcare systems and resolve some of the financial problems associated with teaching basic skills during actual operations. (Bridges et al., 1999)

The new setup has many advantages associated with portability, ease of use and image processing. The small smartphone device has replaced the camera, the video recorder and the monitor. It is a cable-free fully portable hysteroscopic system that can be easily transferred from one location to another, compared to the cumbersome standard tower that is immobilised in a hospital’s operating room. It is easy to perform training courses, even in the pathology department, so it is not mandatory for the uterine specimens to move from one place to another. The high definition camera of the smartphone provides a high-quality image of the uterine cavity, and at the same time, the procedure can be recorded and stored to share in real-time or for future review and analysis. The residents could even use their own smartphone and obtain videos and images of their training hysteroscopies to analyse their technique defects.

In addition, the smartphone, although operating as a stand-alone monitor, has the ability to project the image over a wireless signal to an external monitor, tablet or laptop. To achieve this, a new 4K Apple TV (Apple Inc.), a wireless router, and an iPhone 6s are used, although any iPhone that is capable of running at least iOS 4.3 can be used (Chatzipapas et al., 2016). The real-time video sharing option is very useful for teaching as it allows an interactive discussion about the procedure between the supervisor and the trainees.

After the establishment of the new training programme, residents were able to easily recognise the access and assess the endometrial cavity competently. Due to the realism of the human uterine model, they became familiar with the anatomy and main uterine landmarks. Thus, we anticipate that acquisition of these basic proficiencies will enhance confidence, and mimimise the learning curve in real- life patients in the operating room.

## Conclusion

This is the first report of using a human uterus model for hysteroscopic training. It is also the first time that mobile technology has been applied, and the results are promising. The new low cost portable hysteroscopic setup coupled with human uterine models constitutes a reliable, realistic and potentially effective training system that provides basic hysteroscopic skills outside the operating room with multiple benefits to the residents, healthcare systems and especially the patients.
